# Iron Metabolism: Interactions with Energy and Carbohydrate Availability

**DOI:** 10.3390/nu12123692

**Published:** 2020-11-30

**Authors:** Alannah K. A. McKay, David B. Pyne, Louise M. Burke, Peter Peeling

**Affiliations:** 1 Mary MacKillop Institute for Health Research, Australian Catholic University, Melbourne, VIC 3000, Australia; Louise.Burke@acu.edu.au; 2 Research Institute for Sport and Exercise, University of Canberra, Canberra, ACT 2617, Australia; David.Pyne@canberra.edu.au; 3 School of Human Sciences (Exercise and Sport Science), University of Western Australia, Crawley, WA 6009, Australia; peter.peeling@uwa.edu.au; 4 Western Australian Institute of Sport, Mt Claremont, WA 6010, Australia

**Keywords:** carbohydrate, low energy availability, RED-S, iron deficiency, ketogenic diets, hepcidin, exercise

## Abstract

The provision or restriction of select nutrients in an athlete’s diet can elicit a variety of changes in fuel utilization, training adaptation, and performance outcomes. Furthermore, nutrient availability can also influence athlete health, with one key system of interest being iron metabolism. The aim of this review was to synthesize the current evidence examining the impact of dietary manipulations on the iron regulatory response to exercise. Specifically, we assessed the impact of both acute and chronic carbohydrate (CHO) restriction on iron metabolism, with relevance to contemporary sports nutrition approaches, including models of periodized CHO availability and ketogenic low CHO high fat diets. Additionally, we reviewed the current evidence linking poor iron status and altered hepcidin activity with low energy availability in athletes. A cohesive understanding of these interactions guides nutritional recommendations for athletes struggling to maintain healthy iron stores, and highlights future directions and knowledge gaps specific to elite athletes.

## 1. Introduction

Strategies that support athlete health and training availability are integral to the optimization of training outcomes and competition preparation. Nutrition has been recognized as an important contributor to these goals, with the provision of energy, macronutrients and micronutrients underpinning both health and performance. Energy supply is a basic consideration in sports nutrition, with athletes experiencing both deliberate and unintentional changes to the balance between intake and expenditure as they manipulate body composition and training loads. Although energy balance is the traditional metric by which such changes have been evaluated, the newer concept of energy availability [1] has become a major topic in considerations of athlete health, training consistency and competition performance. Energy availability (EA), calculated by removing the energy cost of an athlete’s exercise program from their dietary energy intake, represents the energy that is remaining to support the body’s normal physiological functioning (e.g., reproductive system, bone metabolism, and endocrine function) [2]. Low energy availability (LEA), arising from reduction in an athlete’s energy intake and/or an increase in exercise load, is associated with downregulation and impairment of key physiological processes due to the lack of adequate energy support [3]. LEA underpins the clinical sequalae associated with the syndromes known as the Female Athlete Triad [4] and Relative Energy Deficiency in Sport (RED-S) [5,6]. While the former focused on disruption to the menstrual cycle and bone health in female athletes, these models now acknowledge that LEA is also an important issue in male athletes [7].

Carbohydrate (CHO) availability has emerged as another key theme of interest, with this term describing the balance between CHO requirements of the muscle and central nervous system (and potentially other organs and body systems) around an exercise session relative to the endogenous and/or endogenous CHO supply [8]. There is plentiful evidence that strategies which achieve high CHO availability (i.e., to balance supply to the demand) are associated with enhancement of exercise capacity and sports performance, particularly during prolonged endurance events requiring high intensity efforts [9,10]. These outcomes have led to recommendations that when optimal performance is desired, endurance athletes adopt strategies of daily CHO intake and/or high CHO availability around key exercise sessions to meet the session fuel demands [8]. However, the application of advanced analytical techniques to investigate exercise–nutrient interactions has shown strategies that achieve low CHO availability (i.e., acute CHO restriction around an exercise session) can amplify cellular adaptations within skeletal muscle during and after exercise [11]. If manipulation of CHO availability could be integrated into the training cycle, matching availability to the demands and goals of each session, a strategic blend of augmented adaptation workouts and targeted quality sessions could lead to enhanced performance outcomes [8,12]. Meanwhile, an alternative approach to metabolic preparation for endurance exercise is to chronically restrict dietary CHO, allowing the muscle to achieve a 2–3 fold increase in fat oxidation, coupled with a simultaneous decrease in CHO utilization, thus shifting its fuel reliance from finite CHO stores to the relatively unlimited body fat reserves [13,14,15]. The overall favorability of these strategies should be considered in the context of an athlete’s performance goals and their requirement for metabolic flexibility [8,16,17]. Furthermore, they should be carefully integrated into the athlete’s periodized training program to meet specific training goals and performance outcomes [8].

Although the main concerns around LEA have targeted reproductive and bone health, there is now greater awareness of the potential for wider disruption to body systems [5,6]. In parallel, there is growing interest in the effects of manipulating CHO availability beyond impacting metabolic changes in the muscle or performance outcomes, to the downstream targets of inter-organ cross-talk. Iron metabolism is emerging as a system that can be influenced by both factors. Poor iron status is often associated with LEA [6], with recent study of 1000 female athletes reporting an odds ratio of 1.64 for a history of anemia, low hemoglobin or low iron stores in those identified with LEA [18]. In addition, there are mechanisms by which exercising under low CHO availability can impair iron regulation [19,20]. Therefore, the purpose of this review is to synthesize the current information on the impact of manipulating energy and CHO availability on iron metabolism, with consideration to current dietary practices adopted by elite endurance athletes. This paper was prepared as a narrative review in recognition of the complexity and the early stage of development of these themes. Our intention is to draw on observations from our own extensive research on each of the separate topics, as well as the work of others, to focus attention on issues that should be further addressed by a systematic series of observational and intervention studies.

## 2. Why Are Adequate Iron Stores Necessary for Athletes?

Iron is fundamentally important to the optimal function of endurance athletes, given the mineral’s role in athlete-relevant processes such as oxygen transport, cellular energy production, cognitive processing, and immune function [21,22]. Compromised iron stores can impair critical physiological processes, with significant negative effects on athlete health and performance. For example, high levels of aerobic fitness, a common prerequisite for elite endurance performance, can be limited by the oxygen-delivery capacity to the muscle [23]. In iron-compromised individuals with anemia, the impairment of hemoglobin production results in decrements to aerobic performance [24]. However, in such cases, once iron stores are restored via oral or intravenous supplementation, increases in VO_2max_ [25], exercise time-trial performance [26] and exercise efficiency [27,28] have been reported.

Despite research and clinical knowledge, iron deficiency in athlete populations remains a common issue. To understand the prevalence, various thresholds used to classify the severity of iron deficiency must be established. Accordingly, three stages of iron deficiency have been proposed: (1) Iron depletion, where iron stores are depleted without hematological consequences; (2) Iron deficiency non-anemia, where erythropoiesis diminishes as the iron supply to the erythroid marrow is reduced; and (3) Iron deficiency anemia, where hemoglobin production falls, resulting in anemia [29]. At a minimum, quantification of serum ferritin, hemoglobin and transferrin concentrations are required to diagnose an iron deficiency, with additional variables such as serum soluble transferrin receptor, hemoglobin mass, or C-reactive protein presenting as potential beneficial adjunct markers of detection [20]. While there is general agreement that iron deficiency can negatively impact performance, there is less conformity surrounding the classification criteria of these categories. Iron deficiency non-anemia has been commonly defined in athletic populations as a serum ferritin of <20 μg·L^−1^ and transferrin <16% [20,29]; however, variations in the literature range from serum ferritin values of <12 μg·L^−1^ through to <40 μg·L^−1^ [29,30,31]. Iron deficiency anemia is thought to be apparent once hemoglobin concentrations become compromised, with diagnostic thresholds below 11.5–12 g·dL^−1^ commonly used. The incidence of iron deficiency non-anemia is reported as 24–47% of female and 0–17% of male athletes [32]; however, rates as high as 86% of female youth athletes from a mixed-sport cohort have been reported [33]. The higher incidence of iron deficiency observed in females has been attributed to the increased iron losses associated with menstruation [34]. However, the high prevalence of low iron stores commonly seen in athletes can also be partially explained by incorporation of iron into new tissues and cells induced by adaptation to training, as well as exercise-associated iron losses via exercise-induced mechanisms such as hemolysis, hematuria, sweating, gastrointestinal bleeding, and acute transient increases in the iron regulatory hormone, hepcidin [35].

## 3. Hepcidin and Iron Regulation

Iron status is tightly controlled in the body by the homeostatic regulation of iron movement across the gut and between cells. Homeostasis is essential, not only to encourage iron uptake in times of need, but also prevent iron toxicity and overload. Iron regulation is governed by the master regulatory hormone, hepcidin, which is released from the liver to dictate the availability of iron for biological functions [36]. The primary action of hepcidin is to bind to, and internalize the body’s cellular iron export channels, ferroportin, located on the cell surface of macrophages of the reticuloendothelial system, enterocytes in the duodenum and hepatocytes [37]. Hepcidin–ferroportin interactions decrease both the amount of iron that can be absorbed from the diet by duodenal enterocytes, and the amount of iron recycled by macrophages. Through this mechanism, hepcidin is able to regulate transferrin and intracellular iron stores in a homeostatic manner. For instance, in iron-deplete individuals, hepcidin concentrations are reduced as a means of encouraging iron absorption to drive the replenishment of iron stores. However, iron excess stimulates liver hepcidin production, in an attempt to prevent further increases in iron supply [38].

While iron status appears to be a dominant factor in hepcidin regulation, inflammation is also known to impact hepcidin levels, and subsequently, iron balance [39]. The inflammatory cytokine interleukin-6 (IL-6) directly stimulates hepcidin production via an increase in signal transducer and activator of transcription 3 (STAT 3) production, resulting in increased transcription of hepcidin from hepatocytes [40]. This mechanism was highlighted through administration of 30 μg·L^−1^ recombinant IL-6, which elicited a 7.5-fold increase in hepcidin concentrations 2 h post-infusion [41]. This outcome was replicated in a study using a 2 ng·kg^−1^ body mass (BM) injection of lipopolysaccharide (i.e., stimulating an inflammatory response), in which IL-6 was increased 3 h post-injection, followed by an increase in hepcidin levels 3 h later (i.e., 6 h post-injection, but 3 h post-IL-6 response) [42]. Since exercise is known to be a potent inflammatory stimulus, the relationship between exercise, inflammation, and hepcidin activity has attracted attention. Indeed, IL-6 is released from the skeletal muscle in response to exercise, playing a key role in mediating the acute phase response [43]. The duration of exercise appears to be the largest determinant of the post-exercise IL-6 response, with increases occurring in a time-dependent exponential manner, peaking immediately post-exercise [44]. Furthermore, exercise intensity and modality can also influence the IL-6 response, with higher intensity and weight-bearing modes (i.e., running vs. cycling) yielding greater increases in post-exercise cytokine levels [43,45]. An early investigation of the link between exercise, IL-6 and hepcidin tracked the time course of changes in these two variables following 60 min of treadmill running (15 min at 75–80% HRpeak, followed by 45 min at 85–90% HRpeak) [46]. Here, a 6.9-fold increase in IL-6 was evident immediately post-exercise, followed by a subsequent peak in hepcidin levels 3–6 h later (5.2-fold increase). This was the first study to demonstrate that increases in hepcidin levels occur subsequent to an exercise-induced inflammatory stimulus. This sequence is important, as it is likely that iron absorption is impaired during the post-exercise period, when hepcidin levels are elevated. This outcome could have negative implications for athletes’ iron balance, particularly when performing frequent high-volume training. Within the exercise literature in particular, hepcidin levels have been used as a surrogate marker of iron bioavailability, with interest in strategies that minimize the hepcidin response to exercise.

Multiple regression analysis of both physiological and biochemical markers has shown that the increase in IL-6 concentrations is a small, yet significant contributor to the magnitude of subsequent hepcidin increase at 3 h post-exercise [47]. Interestingly, nutritional manipulation of the magnitude of the IL-6 response to exercise may provide a mechanism to improve iron absorption during the post-exercise period. Factors that influence the release of IL-6 during exercise include the task duration, mode, intensity, training status, and of importance in the context of sports nutrition, muscle glycogen stores [19,48]. Although an increased production of IL-6 has been demonstrated in response to running or cycling for ≥2 h at moderate to high intensities, this response can be attenuated when CHO is consumed throughout the exercise task to maintain blood glucose concentrations and decrease the reliance on/depletion of muscle glycogen stores [19]. However, studies utilizing exercise bouts <2 h in duration have shown CHO supplementation has minimal impact on IL-6 concentrations, unless exercise is commenced with low muscle glycogen stores, in which case the response is augmented [19]. In this low muscle glycogen scenario, CHO ingestion during exercise bouts of 30–90 min in duration can promote the attenuation of the IL-6 response to exercise [49,50]. Given the relationship between IL-6 and hepcidin activity, an increased IL-6 response resulting from training with low CHO availability and/or low muscle glycogen stores, may increase hepcidin levels 3 h post-exercise, which could then negatively impact iron regulation in athlete cohorts. Therefore, strategies that promote CHO availability may help to limit the post-exercise compromise in iron absorption by attenuating exercise-induced inflammation and subsequently minimizing post-exercise hepcidin levels. However, the quantity and timing of CHO intake are important factors in regulating this response.

## 4. Carbohydrate Availability and Iron Regulation

### 4.1. Post-Exercise Carbohydrate Intake

Post-exercise CHO consumption is an important nutritional strategy to optimize recovery, particularly for endurance athletes. When an athlete’s goal is to maximize post-exercise muscle glycogen restoration to support subsequent training/competition sessions, CHO ingestion should occur as soon as practical after exercise, to take advantage of the higher rates of muscle glycogen synthesis in the early phases of recovery, and maximize the duration of the period when exogenous substrate is available for muscle storage [51]. CHO intake targets for rapid refueling during the 1–4 h following exercise have been set at 1.0–1.2 g·kg·h^−1^, consumed as small regular meals [52]. Several studies have investigated whether such practices also influence iron metabolism. Initial work by Badenhorst et al. [53] examined the effect of consuming 12 mL·kg^−1^ body mass (BM) of a 10% CHO beverage at different stages of recovery following a 60 min interval running task on post-exercise inflammation and hepcidin levels. There were no differences in either IL-6 or hepcidin activity for up to 5 h post-exercise between immediate (15 and 120 min post-exercise) and delayed (120 and 240 min post-exercise) ingestion of CHO [53]. Since peak IL-6 concentrations occur immediately after exercise and return rapidly to baseline within 1–2 h [54], it is likely that this finding reflects the inability of CHO ingestion to affect hepcidin levels when IL-6 concentrations are already elevated. Indeed, similar findings were reported by Dahlquist et al. [55], who investigated the IL-6 and hepcidin response to different post-exercise nutrition support approaches following interval-based cycling sessions (8 × 3 min intervals at 85% of power output at VO_2max_). This study found similar post-exercise IL-6 and hepcidin responses to a recovery beverage containing 75 g CHO and 25 g of protein, the same beverage with the addition of a vitamin D (5000 UI) and vitamin K2 (1000 mcg) complex, or a taste-matched placebo. Therefore, it appears that the post-exercise consumption of CHO occurs too late to influence post-exercise IL-6 or hepcidin levels, raising the prospect that CHO intake may need to occur prior to, or during the exercise session, to be of benefit to this response. Of course, post-exercise CHO consumption should still be emphasized for its other contributions to recovery, such as the restoration of muscle glycogen stores, particularly following strenuous exercise [52].

### 4.2. Carbohydrate Feeding during Exercise

Given the lack of a substantial post-exercise effect from CHO consumption on inflammatory responses and iron regulation, attention turns to the impact of consumption during exercise. Robson-Ansley et al. [56] studied the ingestion of either a 8% CHO solution or a placebo beverage prior to and during a 2 h submaximal run (60% vVO_2max_), at a rate of 2 mL·kg^−1^ BM consumed every 20 min. Immediately following the 2 h run, a 5 km running time-trial was performed. Despite attenuation of the IL-6 response immediately post-exercise when the CHO beverage was consumed, no differences in hepcidin concentration were reported between conditions. Unfortunately, in this study, post-exercise hepcidin concentrations were measured immediately after, and at 24 h of recovery; timings that would likely not reflect the key periods during which hepcidin is elevated (i.e., 3–6 h post-exercise [46]). In a follow-up study, participants ran for 90 min on a motorized treadmill at 75% VO_2peak_, while consuming either a 6% CHO or placebo beverage at a rate of 3 mL·kg^−1^ BM every 20 min [57]. Despite using more appropriate sampling time points (e.g., measuring hepcidin concentrations 3 h post-exercise), no differences in either IL-6 or hepcidin levels were evident between the CHO or placebo beverage trials. One explanation for the absence of an effect is that the selected exercise protocol was too short for muscle glycogen stores to become sufficiently different in terms of eliciting increased IL-6 production. Accordingly, scenarios which involve exercise in the presence of muscle glycogen depletion could elicit a greater influence on iron regulation. On this basis, the manipulation of CHO in the hours prior to exercise is of interest.

### 4.3. Implications of Acute Carbohydrate Restriction

Badenhorst et al. [50] assessed the impact of muscle glycogen stores on iron regulation by implementing an exercise task known to deplete muscle glycogen stores by ~50% [58]. Here, participants performed a 16 km run at 80% vVO_2peak_, followed by 5 × 1 min efforts at 130% vVO_2peak_ with 2 min recovery between efforts. This task was followed by 24 h of diets of either low (3 g kg^−1^ BM) or high (10 g kg^−1^ BM) CHO intake with similar energy support (4100 and 4500 kcal, respectively) [50]. Participants then performed an interval-based running task (8 × 3 min at 85% vVO_2peak_) 24 h later, with IL-6 and hepcidin concentrations measured pre/post-exercise and at 3 h post-exercise, respectively. The results showed that the high CHO trial was associated with an attenuated post-exercise IL-6 response (2-fold vs. 3-fold increase) with a trend towards lower hepcidin levels 3 h post-exercise compared to low CHO trial (4.1 vs. 6.4 nM; d = 0.72). While differences in the hepcidin response did not reach statistical significance, this study demonstrated the potential for a moderately greater increase in hepcidin following exercise undertaken with theoretical (albeit not quantified) depletion of muscle glycogen stores. This study provides some evidence of an association between macronutrient intake, inflammation and iron regulation, especially when considered in the context of the athlete’s pre-exercise nutritional state.

These outcomes become important in light of the contemporary interest in strategies to periodize CHO availability around specific training sessions. It is likely that periodic glycogen depletion occurs within the training programs of high performance endurance athletes, with the high frequency and load (intensity and volume) of their sessions preventing a full glycogen restoration between each workout. However, more recent practices that deliberately manipulate low CHO availability have evolved as a strategy to enhance endurance training adaptations. For example, in a survey of the self-reported practices of elite runners and race walkers, a majority (62%) of the distance athletes reported training in a fasted state, typically 1–3 times/week around easier sessions [59], where low liver glycogen stores and low exogenous CHO availability would be expected [8]. Although some athletes identified “practical” reasons for this behavior (e.g., to allow them to get more sleep before training or to reduce the risk of gut discomfort during training), the majority identified a strategic rationale for the approach (e.g., to assist with body composition changes or enhance the training response). Furthermore, 44% of the total cohort reported an occasional restriction of CHO intake around or between some training sessions, theoretically creating a scenario of training with low muscle glycogen availability, which would upregulate the accompanying cell signaling and gene expression response [60]. Although many of the athletes who reported such practices identified that they were underpinned by a strategic rationale, a significant number of the group who identified the absence of CHO restriction strategies noted a lack of overall performance improvement, or an increase in illness/injury, among their experiences [59]. Indeed, given the potential exacerbation of inflammation and hepcidin activity following exercise with low muscle glycogen stores, the implications to iron balance become an interesting point for consideration.

Recent work from our group investigated this question of dietary manipulation in an applied setting, with elite triathletes performing four 48 h manipulations of diet and exercise [61]. Here, two trials involved a ‘train-high, sleep-low, train-low’ sequence [8], which restricted CHO intake between the two training sessions to achieve low glycogen training on the session of interest. The remaining two trials involving exercise performed under consistently high CHO availability (8 g kg BM^−1^ day^−1^ CHO). The final session of the sequence involved a 45 min running protocol, or a 60 min cycling protocol, with the cycling trials of higher intensity (evidenced by increased heart rate and RER). During the running trials, no differences in hepcidin concentrations were evident between conditions of high or low CHO availability. However, during cycling trials, when exercise intensity was increased, a ~72% greater hepcidin response was evident during the ‘train-high, sleep-low, train-low’ dietary condition. Taken together, it appears that muscle glycogen availability and exercise intensity are both critical factors in determining the magnitude of the post-exercise hepcidin response, and that alterations in iron regulation may only occur once a critical level of metabolic stress is achieved. An important practical outcome of this study was the demonstration that strategies of acute CHO periodization can be implemented throughout the training cycle without altering iron regulation if applied to sessions of short duration and low intensity. This outcome supports previous suggestions to maximize the efficacy of targeted approaches [8], where low CHO availability should be periodized around lighter training sessions to enhance the molecular adaptations to training, while high CHO availability can be used to support training when quality and/or high intensity are required.

### 4.4. Implications of Long Term Carbohydrate Manipulation

Although it may be possible to integrate acute restriction of CHO into the training program with minimal influence on post-exercise iron regulation, longer-term manipulation of CHO can yield unavoidable or larger alterations to iron metabolism, which, over time, could eventually deplete iron stores with negative effects to an athlete’s health, wellbeing and performance. To explore this chronic effect, Badenhorst et al. [62] had trained endurance runners complete two structured 7 day training blocks while consuming diets of either low (3 g·kg^−1^ BM) or high (8 g·kg^−1^ BM) CHO content. On days 1 and 7 of each training week, athletes performed a 45 min treadmill run at 65% VO_2max_ to measure the IL-6 and hepcidin response to exercise. Contrary to expectations, there were no clear differences in post-exercise IL-6 or hepcidin concentrations between Days 1 and 7, or between dietary conditions. However, interrogation of the study protocol notes that it scheduled five days of key training sessions, followed by a rest day on Day 6, then the post-intervention test on Day 7. As such, it is possible that the day of rest, combined with a higher (22%) protein intake in the isocaloric low CHO condition, could have allowed both diets to achieve sufficient restoration of muscle glycogen on Day 7. Consequently, any differences in inflammatory and iron regulatory markers to the observed exercise session were likely negated.

The ketogenic low CHO high fat (LCHF) diet represents another model of chronic CHO restriction of current interest. Ketogenic diets are characterized by CHO intakes of <50 g·day^−1^ and low to moderate protein intake (~15% energy intake), with the remaining daily energy consumed in the form of dietary fat [63]. Adherence to a LCHF diet increases blood ketone concentrations, while re-tooling the muscle to substantially increase fat oxidation, including an increase in the exercise intensity at which maximal rates of fat oxidation occur [17,63]. Studies of medium term (e.g., 4 weeks) adherence to such diets show reduced resting muscle glycogen content and reduced utilization of glycogen during exercise [64]. It is of interest to note that even with extreme restriction of dietary CHO intake, gluconeogenesis and the storage of muscle glycogen is enabled from precursors such as lactate, glycerol and some amino acids [17,64]. Of note, the time course of adaptation to a LCHF diet remains a contentious issue, largely due to the absence of a clear definition of the processes and outcomes of keto-adaptions. Although it has been argued that 2–3 months, or even longer, may be needed for complete keto-adaptation to occur [17], substantial alterations in substrate utilization and ketone concentrations have been demonstrated in as little as 5 days adherence to a ketogenic LCHF diet [15,65]. Controversially, and of importance to the current review, it has been proposed that long-term adherence to a ketogenic diet enhances glycogenesis, restoring or “normalizing” muscle glycogen content to levels similar to that of athletes adhering to high CHO diets [66]. However, this theory is only supported by evidence from a single cross-sectional study, where endurance athletes who reported (and had confirmed) long-term adherence to a ketogenic LCHF diet (>6 months; <50 g·day^−1^ CHO), were compared to a similar cohort who consumed diets with higher CHO availability [66]. Meanwhile, studies with similar methodology [67] and other lines of interrogation contradict this theory, justifying that further scrutiny is warranted [17]. Nevertheless, with the majority of studies supporting the notion that ketogenic LCHF diets are associated with chronically reduced muscle glycogen content, it is possible that such nutritional approaches can result in a cumulative increase in hepcidin levels (both at baseline and post-exercise), with negative implications to iron status. Of course, there may also exist other iron-related issues from such diets, on the basis of the food choice changes, which would impact the quantity and sources of dietary iron intake.

To explore such issues, our group recently examined the effect of 3 weeks adherence to a LCHF diet (<50 g·day^−1^ CHO, ~80% fat) during a period of intensified training on iron metabolism in elite race walkers [68]. The dietary iron content of the LCHF diet was ~25% lower than that of the CHO-rich diet (13.7 vs. 17.8 mg·day^−1^; *p* = 0.005) due to the exclusion of fortified grains and cereals, which provide a substantial source of non-heme iron in the Western diet. Despite a lower dietary iron intake, the LCHF group exhibited a smaller decrement in serum ferritin levels (23% decrease) than athletes adhering to CHO-rich diets (37% decrease; *p* = 0.021) [68]. While this outcome seems contradictory, we propose that the greater decrease in serum ferritin may reflect a larger, more adaptive hematological response to training in the group exposed to consistently or strategically high CHO availability. Here, iron may have been used for adaptive processes such as increases in the production of hemoglobin or iron-associated enzyme activity, outcomes that benefits aerobic performance. Indeed, these athletes experienced a mean 4.8–6.0% improvement in 10 km race walk performance, as compared to the 1.6–2.3% decrement evident in athletes adhering to a LCHF diet [13,14]. However, there are multiple factors that can influence performance, and unfortunately, hematological adaptation (i.e., hemoglobin mass) was not quantified to confirm this hypothesis. Another investigation of moderately trained individuals (defined as endurance training >7 h per week), assessed the impact of a 12-week LCHF dietary intervention on hematological parameters [69]. In a free-living situation where participants self-selected all their foods, athletes adhering to the LCHF diet consumed significantly less dietary iron than athletes adhering to a high CHO diet (12.0 vs. 18.2 mg·day^−1^). However, in this instance no changes in serum ferritin were evident after 12 weeks in either the high CHO or LCHF dietary groups. Differences between our work and that of McSwinney and colleagues [69] may be attributed to the caliber of athlete and level of the training stimulus. It is likely that the elite athlete cohort from our work completed a more demanding training schedule than the moderately-trained participants engaged by McSwinney et al. [69], which in combination with the highly hemolytic nature of race walking, may have elicited larger exercise-associated iron losses [35], and therefore, reductions in iron stores.

We also studied the impact of a LCHF diet on the iron regulatory response to exercise [68]. Here, a greater IL-6 and hepcidin response occurred following a 25 km exercise protocol in athletes who had adapted to a LCHF diet, compared to athletes that remained on a high CHO diet. This result presents the possibility that iron absorption may have been impaired in keto-adapted athletes in the hours following exercise. Importantly, however, the differences in serum ferritin levels need to be considered, as they have a strong homeostatic influence on the magnitude of the post-exercise hepcidin increase [47,70]. Accordingly, the higher serum ferritin levels evident in the LCHF group post-intervention, may have contributed to the greater hepcidin outcomes reported at 3 h post-exercise. This question prompted examination of the iron regulatory response in a subset of athletes, matched for serum ferritin levels, to remove any influence of baseline iron status [71]. This follow-up study revealed no differences in post-exercise hepcidin concentrations between keto-adapted athletes (<50 g·day^−1^ CHO) and those adhering to CHO-rich diets (~8·g·kg^−1^ BM·day^−1^ CHO). Therefore, it collectively appears that iron status may have been a confounding factor in our initial study [68], and that an athlete’s initial iron status may exert a more dominant influence over hepcidin expression than dietary manipulation. In conclusion, while acute studies manipulating muscle glycogen content can alter hepcidin activity, evidence of altered iron regulation resulting from chronic CHO restriction is yet to be clearly demonstrated. It is possible that prolonged adherence to a low CHO diet may result in an adaptive state, whereby these acute alterations subside as the dietary adherence is maintained over time. However, this assertion is speculative, and future research is required to confirm this prospect.

Finally, given the potential for negative implications to iron metabolism when training with low CHO availability, it was speculated that sustained high CHO availability may exert a positive influence on iron metabolism. A recent investigation had elite race walkers adhere to a novel dietary strategy aimed at optimizing endogenous and exogenous CHO availability for 2 weeks [72]. This dietary approach strategically incorporated a number strategies to promote high CHO availability, which included high CHO intake (10–12 g·kg^−1^ BM), gut training strategies [73], low residue foods, and sucralose ingestion. The combination of such strategies was intended to increase CHO availability and oxidation during prolonged high intensity exercise, in an attempt to improve exercise economy and gut tolerance [74], whilst positively influencing athletic performance. However, these outcomes may also improve the ability to better sustain blood glucose concentrations and muscle glycogen stores, which could attenuate the IL-6 response to exercise and minimize post-exercise hepcidin levels. Regardless, no differences in serum ferritin, inflammation or hepcidin concentrations were evident in athletes adhering to the novel, very high CHO dietary approach, compared to athletes consuming a more moderate CHO intake (6–8 g·kg^−1^ BM). Therefore, it appears that there is no additional benefit to iron regulation from increasing CHO intake to very high levels, leaving us to conclude that a moderate CHO intake appears sufficient to mediate the various factors we know have an impact on iron regulation.

## 5. Energy Availability and Iron Regulation

While CHO has received significant attention as a potential moderator of iron metabolism, a more recent focal point has been the impact of inadequate energy availability. LEA in athletes, which can arise from restrictions in energy intake, excessive energy expenditure, or a combination of both, is thought to impair key physiological processes that underpin health and performance [6]. Interestingly, it has been suggested that low iron stores may contribute to LEA or its clinical manifestations, yet it is also acknowledged that LEA may itself contribute to low iron status in athletes [75]. With no innate mechanism available for the body to synthesize iron, humans are solely reliant on dietary iron sources to replace incidental daily [76] and exercise-induced [35] iron losses, including the replacement of iron used for adaptive purposes. Absolute restriction of energy intake, which is commonly involved in scenarios of LEA in weight restricted or weight sensitive sports [77], may contribute to reduced intake of micronutrients, exacerbated by disordered eating or other restrictions of dietary range. Furthermore, scenarios of LEA can also be accompanied by very high energy expenditures resulting from excessive training loads, which potentially increase exercise-induced iron losses via mechanisms such as hemolysis, sweating, gastrointestinal bleeding, inflammation and hepcidin elevations [35]. This mismatch between iron losses and iron intake may partially explain the high rate of iron deficiency commonly observed in athletes with LEA [18].

The action of the iron regulatory hormone hepcidin may also be influenced by LEA. Increases in resting hepcidin concentrations have been reported in physically active military personnel completing a 4 day military training exercise, that elicited a 55% energy deficit and 2.7 kg decrease in body mass (EI = ~2200 kcal·day^−1^; EE = ~6100 kcal·day^−1^) [78]. Here, the increase in resting hepcidin levels seen was positively associated with energy expenditure (*r* = 0.40), and negatively correlated with energy balance (*r* = −0.43). However, the macronutrient content of the diet had no influence on either IL-6 or hepcidin levels. Collectively these data indicate a link between hepcidin expression and energy provision, occurring independently of an inflammatory stimulus, highlighting the importance of maintaining energy availability to avoid unnecessary elevations in hepcidin concentrations. Similar findings were reported in a crossover study of highly trained endurance athletes, where resting hepcidin concentrations were increased by a 3 day exposure to LEA (18 kcal·kg·FFM^−1^) compared to a diet of sustained adequate energy availability diet (52 kcal·kg·FFM^−1^) [79]. In this same study, 75 min of treadmill running at 70% VO_2max_ yielded a significantly larger post-exercise IL-6 response in the LEA trial, compared to the adequate EA condition. Here, a steady decline (−28%) in muscle glycogen content was evident over the 3-day LEA period, which is likely responsible for the augmented inflammatory response reported. However, despite differences in IL-6 between dietary conditions, no significant differences in hepcidin levels at 3 h post-exercise were reported. Taken together, the differences in resting hepcidin concentrations between dietary conditions (without changes to inflammation), in addition to a similar post-exercise hepcidin increase (occurring despite differences in IL-6 levels), provides further evidence that LEA may be influencing hepcidin activity via a non-inflammatory mechanism that is independent of the STAT-3 pathway primarily responsible for CHO induced alterations in hepcidin levels [40].

The precise mechanism underpinning the observed alterations to hepcidin concentrations associated with LEA are still in question. One prospect is the regulation of hepcidin via gluconeogenic signaling [80]. For instance, in response to metabolic disturbances induced by food deprivation, a ~5-fold increase in transcription of the hepcidin gene (hepcidin antimicrobial peptide; HAMP) has been reported. This upregulation was followed by a ~2-fold increase in hepcidin concentrations, attributed to activation of cyclic adenosine monophosphate response binding protein (CREBH) [80]. This study demonstrated the ability for hepcidin to be a gluconeogenic sensor during times of starvation, potentially making this pathway a candidate to explain the increased hepcidin expression in athletes with LEA. Alternatively, the expression of another key regulator of iron metabolism, erythroferrone (ERFE), can also be regulated by nutrient availability [81]. Increases in ERFE act as an inhibitor of hepcidin expression, and therefore, ERFE reductions that occur in response to starvation result in an increase in hepcidin concentrations [81]. Irrespective of the current lack of knowledge surrounding the precise mechanistic pathway relevant here, the increase in hepcidin levels that are evident in scenarios of LEA has led to the proposition that hepcidin could be a useful biomarker for early identification of LEA in athletes [82]. Although this is an intriguing prospect, it is noted that the majority of evidence supporting this association has been largely drawn from animal studies, or models of starvation. Therefore, it remains to be determined whether LEA in athletes creates metabolic perturbations of sufficient magnitude to alter these signaling pathways. Accordingly, future research should identify the exact mechanism(s) responsible for alterations in hepcidin expression caused by LEA before it can be considered as a useful biomarker in the early identification of this issue in athletes.

Interestingly, it is also possible that hepcidin concentrations may be indirectly affected by LEA as a secondary response to other hormonal perturbations. A study of exercising military personnel had participants placed into a 55% energy deficit for 28 days, while receiving either a weekly 200 mg testosterone injection, or an isovolumetric placebo [83]. Participants receiving placebo injections reported no changes to serum ferritin or hepcidin levels over the 28-day energy deficit period, however, a decline in hemoglobin concentrations and erythropoiesis was noted. In comparison, participants that were supplemented with testosterone reported a 41% decline in hepcidin levels, which increased iron availability to support a 34% increase in erythropoietin concentrations, allowing erythropoiesis and hemoglobin concentrations to be sustained during the energy deficit. These outcomes indicate that testosterone has suppressive effects on hepcidin expression, which is particularly important in scenarios of LEA where hepcidin may otherwise be elevated. Moreover, the key female sex hormone, estrogen, can have a similar suppressive effect on hepcidin concentrations [84,85]. Such findings become interesting in the context of athletes, where low sex hormone concentrations are a common outcome of sustained LEA and part of the clinical sequalae of RED-S [7,86]. Therefore, an increase in hepcidin expression may indirectly occur in response to declining estrogen or testosterone concentrations, which may subsequently implicate poor iron stores as a secondary outcome of LEA.

**Figure 1:**
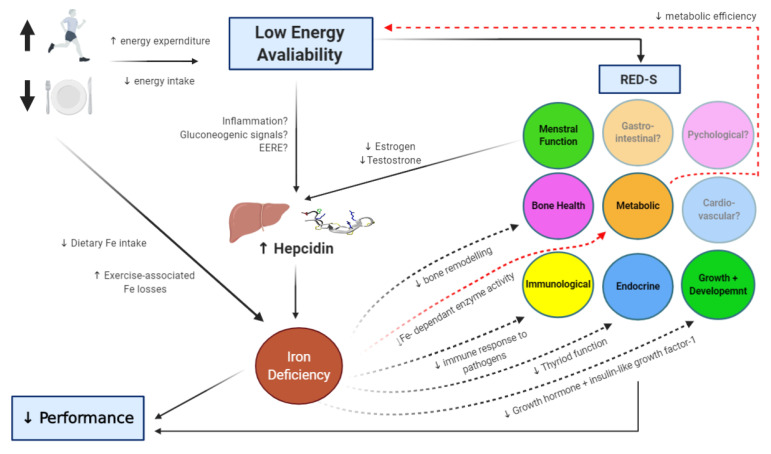
Schematic representation of the interactions between low energy availability (LEA) and iron status in athletes. Solid lines identify pathways where LEA is thought to affect iron status, often mediated by hepcidin expression. Broken lines indicate how iron deficiency can exacerbate other health consequences associated with LEA in relation to the Relative Energy Deficiency in Sport syndrome (RED-S) [6].

While we have primarily reviewed mechanisms by which LEA can augment hepcidin levels, potentially leading to a state of iron deficiency, there is also evidence of a bi-directional relationship in which low iron stores contribute to an energy deficit (Figure 1). For instance, the oxidative production of adenosine triphosphate (ATP) through the electron transport chain requires non-heme iron sulphur enzymes and heme-containing cytochromes [22]. In cases where iron stores are compromised, an athlete may become metabolically inefficient, characterized by a shift from ATP production via oxidative phosphorylation towards anaerobic metabolism. This effect increases the energy expenditure for a given exercise task, potentially reducing energy availability [6]. Additionally, low iron stores may exacerbate some of the other negative health outcomes associated with LEA (Figure 1) [75]. For example, associations between iron status and bone mineral density have been reported in non-athletic populations [87], leading to the assertion that chronic iron deficiency can induce bone resorption [88]. Impairments in bone turnover markers have been demonstrated in studies where LEA has been induced [89,90], which over time, may lead to poor bone health. In athletes with both depleted iron stores and LEA, the negative impact on markers of bone turnover may be amplified, potentially accelerating the progression towards undesirable and irreversible conditions such as osteopenia. Another key system possibly influenced by LEA is the immune system [6], with evidence of an increased incidence of illness reported in athletes with LEA [91]. However, it has also been proposed that LEA has minimal impact on immunity, and studies demonstrating this association may instead be mediated by poor mental health (e.g., anxiety or perceived stress) [92], another proposed consequence (and cause) of LEA in athletes [6]. Nevertheless, iron plays an important role in mounting an effective immune response to invading pathogens, and iron deficiency may contribute to decreased immune resistance and increased susceptibility to infection [93]. It may be that when LEA and depleted iron stores occur simultaneously in athletes, that immune resistance is further compromised, potentially via the indirect effect of LEA on psychological health, which is known to impact immune resistance [94]. Finally, iron deficiency can reduce thyroid functioning [95], and decrease the release of growth hormone and insulin-like growth factor [96]. These alterations (which also occur in scenarios of LEA to preserve energy [6]) can have wide-reaching effects, including interfering with growth, reproduction, bone health, and metabolism [75,97]. Accordingly, it may be that the identification of an iron deficiency can serve as an early indicator of LEA, and therefore, dieticians working with athletes wishing to correct an iron deficiency might also consider screening for LEA and clinical signs of RED-S. Furthermore, correcting an iron deficiency may be an important first step in minimizing some of the other negative health consequences that can result from LEA.

## 6. Conclusions

It appears that nutrient availability can impact the iron regulatory response to exercise. With regard to CHO availability, acute manipulation of muscle glycogen content, which causes the athlete to “train low”, appears to increase hepcidin levels during the recovery from exercise. Therefore, athletes who wish to integrate this specialized training strategy into a periodized training/nutrition program should focus the nutrient manipulation on training sessions that are low in intensity and short in duration to minimize any potential influence on hepcidin concentrations and iron regulation. To date, chronic investigations of CHO restriction (i.e., ketogenic LCHF diets) have not shown clear evidence of negative effects on either iron status or iron regulation. However, dietary iron content is typically lower in LCHF menus as compared to that of CHO-rich diets, which should be considered when athletes are adopting these approaches long term. As for the impact of energy availability, investigations in animal models and of military personnel indicate a link between LEA and iron metabolism; however, studies to date in athletes are limited. Accordingly, future research should be directed towards understanding the effects of energy deficit (both acute and chronic) on hematological functions, and well as their interaction with other health systems.
